# Genome-Wide Identification, Characterization, and Expression Analysis of the NAC Transcription Factor in *Chenopodium quinoa*

**DOI:** 10.3390/genes10070500

**Published:** 2019-06-30

**Authors:** Feng Li, Xuhu Guo, Jianxia Liu, Feng Zhou, Wenying Liu, Juan Wu, Hongli Zhang, Huifen Cao, Huanzhen Su, Riyu Wen

**Affiliations:** 1 College of Life Science, Shanxi Datong University, Datong 037009, China; 2 Research and Development Center of Agricultural Facility Technology, Shanxi Datong University, Datong 037009, China; 3 Maize Research Institute, Shanxi Academy of Agricultural Sciences, Xinzhou 034000, China

**Keywords:** quinoa (*Chenopodium quinoa*), NAC transcription factor family, phylogenetic analysis, gene expression patterns

## Abstract

The NAC (NAM, ATAF, and CUC) family is one of the largest families of plant-specific transcription factors. It is involved in many plant growth and development processes, as well as abiotic/biotic stress responses. So far, little is known about the NAC family in *Chenopodium quinoa*. In the present study, a total of 90 *NACs* were identified in quinoa (named as *CqNAC1-CqNAC90*) and phylogenetically divided into 14 distinct subfamilies. Different subfamilies showed diversities in gene proportions, exon–intron structures, and motif compositions. In addition, 28 *CqNAC* duplication events were investigated, and a strong subfamily preference was found during the *NAC* expansion in quinoa, indicating that the duplication event was not random across *NAC* subfamilies during quinoa evolution. Moreover, the analysis of Ka/Ks (non-synonymous substitution rate/synonymous substitution rate) ratios suggested that the duplicated *CqNACs* might have mainly experienced purifying selection pressure with limited functional divergence. Additionally, 11 selected *CqNACs* showed significant tissue-specific expression patterns, and all the *CqNACs* were positively regulated in response to salt stress. The result provided evidence for selecting candidate genes for further characterization in tissue/organ specificity and their functional involvement in quinoa’s strong salinity tolerance.

## 1. Introduction

Quinoa (*Chenopodium quinoa* Willd., 2n = 4× = 36) is an annual crop that originates from the Andean region of South America [1]. It is recognized as a crop of great value for its high nutritious content and high abiotic stress tolerance [2]. Quinoa produces nutritious grains, which are gluten-free, and have an exceptional balance between oil, essential amino acids, carbohydrates, minerals, vitamins, and dietary fibers [2,3]. Meanwhile, it has a good tolerance to high salinity, drought, and frost, which help it thrive under adverse climatic and soil conditions [4,5,6]. Due to its potential health benefits resulting from the outstanding nutritional value of seeds, and the great adaptability to adverse environments displayed, the emerging crop was recognized by the United Nations when 2013 was declared the International Year of Quinoa [7,8]. Until now, an increasing number of researchers have devoted themselves to the study of quinoa, and a draft of the *C. quinoa* genome sequence was recently reported [8,9], which provided insights into the mechanisms underlying agronomically important traits of quinoa and laid the foundation for accelerating the genetic improvement of other crops.

Transcription factors are a group of proteins which can regulate the expression of target genes to control plant biological processes. The NAC (NAM, ATAF, and CUC) family is one of the largest families of plant-specific transcription factors [10,11]. Members in the family are characterized by a highly conserved N-terminal region (NAC domain) and a relatively divergent C-terminal transcriptional activation region (TAR) [12,13]. The NAC domain is involved in DNA binding and can be further divided into five subdomains labeled from A to E, while the TAR region usually is related to the regulation of diversity, and may determine some specific functions [10]. The NAC family widely exists in various kinds of plants and is involved in many plant growth and development processes, including lateral root formation [14], leaf senescence [15], flower morphogenesis [16,17], fruit ripening [18], seed development [19], secondary wall synthesis [20], and hormonal signaling [21]. In particular, *NAC* genes respond to many abiotic/biotic stresses, such as high salinity [22], drought [23], temperature [24], and pathogen defense [25]. Thus, *NAC* genes are important for various plants to withstand adverse environmental conditions. Soil salinization is a major threat to agriculture and causes a substantial reduction in crop yield and quality. Since quinoa has been characterized as a highly salt-tolerant crop, the mechanism of tolerance has been studied, and novel genes which contribute to salinity tolerance were identified [4,26]. 

Members of the *NAC* family have been identified in various plant species, such as *Arabidopsis* [10,27], rice [28], foxtail millet [13], grape [29], potato [30], maize [31], Chinese cabbage [32], cassava [33], tomato [34], melon [35], switchgrass [36], woodland strawberry [37], cotton [12], and bread wheat [38]. However, genome-wide analysis of the *NAC* family in quinoa is lacking. With quinoa genome sequencing completed, an opportunity was opened to investigate gene families in quinoa, and a systematic research of the *NAC* family in quinoa is expected. In the present study, we identified putative *NAC* genes in quinoa and carried out a relatively detailed study of their phylogeny, genomic structures, conserved motifs, expansion patterns, and expression profiles. Our results will lay an important foundation for future research on the molecular evolution and biological function of the *NAC* family in quinoa.

## 2. Materials and Methods

### 2.1. Sequence Retrieval and Structural Analysis

The quinoa genome database (v1.0) was downloaded from Phytozome v12 (https://phytozome.jgi.doe.gov/pz/portal.html). The *Arabidopsis* NAC amino acid sequences were obtained from TAIR (http://www.arabidopsis.org) and were used as queries by searching against the quinoa genome database using the BLASTP program with default parameters [39]. Afterward, the conserved NAC domain of all the potential quinoa NACs were confirmed in the Conserved Domain Database in National Center for Biotechnology Information (NCBI) (https://www.ncbi.nlm.nih.gov/). Finally, quinoa NAC sequences with at least four out of five conserved NAC subdomains were selected for the following analysis [10]. Primary and secondary protein structures were predicted with ProtParam (http://web.expasy.org/protparam/) and SOPMA (https://npsa-prabi.ibcp.fr/cgi-bin/npsa_automat.pl?page=/NPSA/npsa_sopma.html). The subcellular localization was inferred with PSORT (https://psort.hgc.jp/form.html) and Cello (http://cello.life.nctu.edu.tw/).

### 2.2. Phylogenetic Analysis, Gene Structure, and Protein Motif Prediction

All putative NAC sequences in this study were aligned using the ClustalW program [40]. The alignment file was then used to construct neighbor joining (NJ) phylogenetic trees by MEGA7 with 1000 bootstrap iterations [41]. The information of each *NAC* gene in quinoa was retrieved from the quinoa database, and the genomic schematic diagrams of the *NACs* were obtained by comparing the genomic sequences and their predicted coding sequences using the GSDS tool (http://gsds.cbi.pku.edu.cn/). The conserved motifs of the quinoa NAC proteins were identified using the MEME program (http://meme-suite.org/tools/meme) with parameters set based on a previous study [42]. Sequence logos of the conserved domains were generated with the WebLogo program (http://weblogo.berkeley.edu/).

### 2.3. Chromosomal Localization and Gene Duplication

The chromosomal positions of the quinoa *NAC* genes were searched from the quinoa database. Duplicated gene pairs were investigated as described in previous reports [43] and illustrated with the Circos program [44]. The Ka (non-synonymous substitution rate) and Ks (synonymous substitution rate) were estimated by DnaSP v5.0 software [45], and the selection pressure for each duplicated gene pair was calculated by the Ka/Ks ratio.

### 2.4. Plant Materials, RNA Extraction, and Quantitative Real-Time PCR

Sterilized quinoa seeds were grown in a growth chamber at 24 °C/22 °C day/night with a photoperiod of 16 h. RNA samples were collected when seedlings were at the 4–5 leaf stage. Root, stem, leaf samples, and the root samples exposed to 300 mM NaCl (salt stress) for 0, 1, and 3 h, were harvested and were immediately frozen in liquid nitrogen and stored at −80 °C until RNA isolation. The root samples exposed to 300 mM NaCl for 0 h were selected as controls. Total RNA of each samples (100 mg) was extracted using the RNeasy Plant Mini Kit (QIAGEN), and cDNA was prepared using SuperScript™ III Reverse Transcriptase kit (Invitrogen). The quantitative real-time PCR (qRT-PCR) primers were designed (Table S5) and synthesized commercially (HUADA Gene, Beijing, China). The qRT-PCR analysis was performed in the ABI ViiA 7 Real-Time PCR system (Applied Biosystems, USA) by 2× QuantiTect SYBR Green PCR mix (QIAGEN), with the programme of 40 cycles and an annealing temperature of 60 °C. The quinoa Elongation Factor 1 alpha (*EF1α*) gene was used as an endogenous control. Relative gene expression levels were calculated using the 2^−⊿⊿Ct^ method [46]. Each experiment was repeated in triplicate using independent RNA samples. The expression profiles of the *NACs* in quinoa were clustered using the Cluster 3.0 software [47]. 

## 3. Results

### 3.1. Genomic Identification of Putative NACs in Quinoa

In this study, we identified a total of 90 putative *NAC* genes in quinoa and designated them as *CqNAC1*–*CqNAC90* (Table S1). The protein structures of CqNACs were highly diverse, the amino acid numbers of all identified CqNACs varied from 145 (CqNAC28) to 631 (CqNAC16), and molecular weight ranged between 16.9 (CqNAC28) and 70.2 kDa (CqNAC16). The isoelectric points ranged from 4.60 (CqNAC86 and CqNAC87) to 9.61 (CqNAC29).

### 3.2. Phylogenetic Analysis of the NAC Family in Quinoa

**Figure 1:**
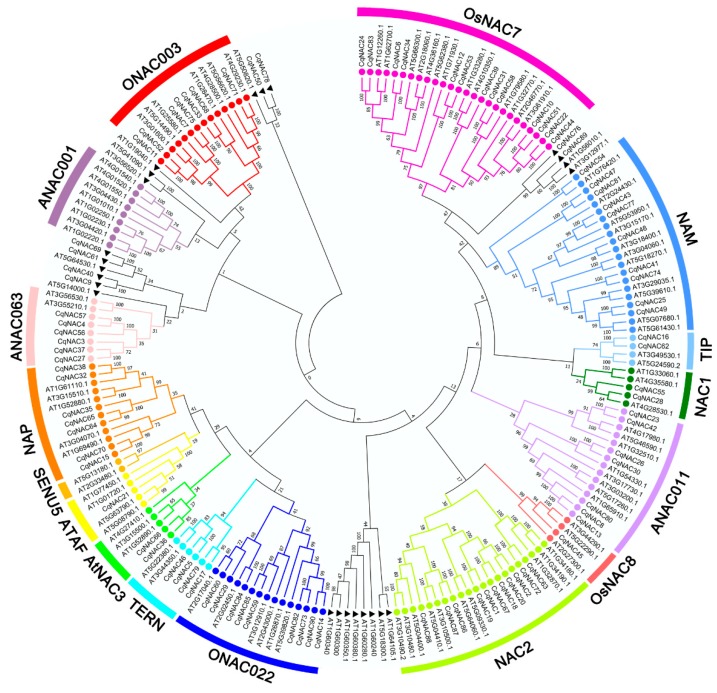
Phylogenetic relationships of the *NAC* family in quinoa and *Arabidopsis*. Full-length amino acid sequences were aligned using ClustalW, and the phylogenetic tree was constructed using the MEGA7 method. The subfamilies are labeled and indicated with different colors. The *NACs* labeled with a black triangle indicate no group members. The numbers at the nodes represent bootstrap support values from 1000 replicates.

**Figure 2:**
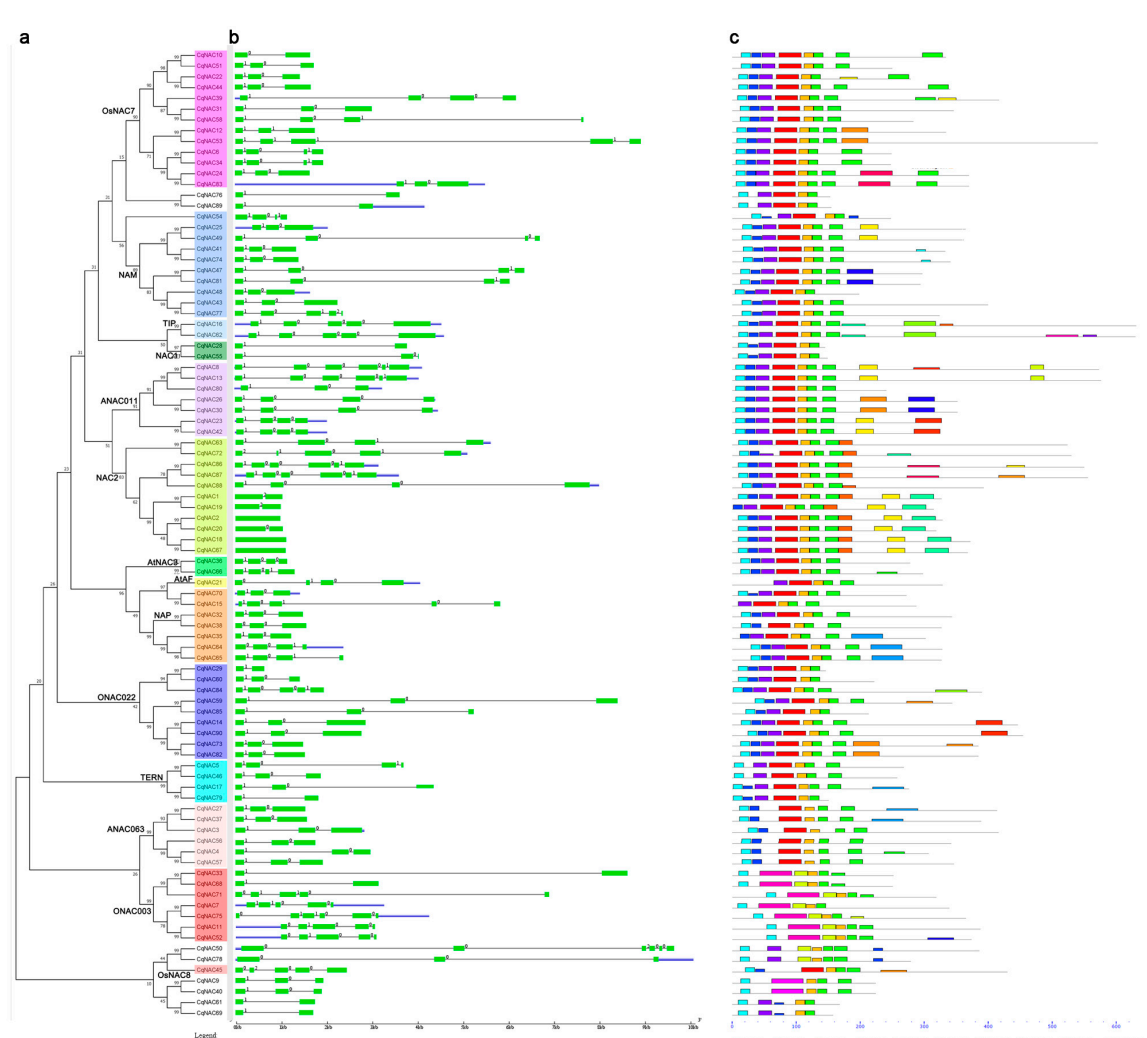
The gene structures and conserved motifs of *CqNAC* genes according to the phylogenetic relationship. The phylogenetic tree (**a**) was generated using the MEGA7 method. Gene structures of *CqNACs* (**b**) were predicted with GSDS software. The exons are represented by green boxes, and the introns are indicated by black lines. The conserved motifs (**c**) were identified by the MEME web server. Each motif is indicated by a colored box numbered at the bottom. The location of each motif can be estimated using the scale at the bottom.

We performed the phylogenetic analysis of the identified *CqNAC* genes by using MEGA7 (Figure 1, Figure 2a, Table S2). The *CqNAC* subfamily was defined by a previous classification in the phylogenetic analysis [10]. In this study, the results indicated that 90 *CqNACs* can be divided into 14 subfamilies, each of which contained a different percentage of the gene members (Figure S1). The OsNAC7 subfamily (14%) contained the most members, followed by the NAC2 (12%) and NAM (11%) subfamilies. The least represented subfamilies were the OsNAC8 (1%) and ATAF (1%) subfamilies. Additionally, no *CqNACs* were found in the ANAC001 and SENU5 subfamilies, suggesting that they might have been lost in these subfamilies.

### 3.3. Exon/Intron Structures and Conserved Protein Motifs of CqNACs

Since the overall pattern of intron position acted as typical imprints of the evolution within some gene families [48,49], the gene structures and intron phases were investigated in the *CqNAC* family to obtain further insight into their evolutionary path (Figure 2b, Table S2). Results showed that most of the *CqNACs* (87 of 90 *CqNACs*) contained introns, the numbers of introns varied from 1 to 5, and the main gene structure was three exons and two introns (36 of 90 *CqNACs*). Three *CqNACs* (*CqNAC2*, *CqNAC18*, and *CqNAC67*) from the NAC2 subfamily had no intron, while *CqNAC8* (ANAC011 subfamily), *CqNAC13* (ANAC011 subfamily), *CqNAC50* (No group), *CqNAC75* (ONAC003 subfamily), *CqNAC86* (NAC2 subfamily), and *CqNAC87* (NAC2 subfamily) contained the maximum number of introns. The gains and losses of introns can result in diversity of gene structure. As shown in Figure 2b, some OsNAC7 and NAC2 subfamilies lost an intron between the first and second exons, and the ONAC022 subfamily lacked an intron between the second and third exons. A gain of an intron in the third exon was observed in OsNAC7, NAP, and NAM subfamilies. By contrast, no change was observed in the gene structure of the TIP and ANAC063 subfamilies.

To further investigate the structural diversity of putative NAC proteins in quinoa, 20 distinct conserved motifs were revealed by the MEME program. The motif distribution corresponding to the phylogenetic tree of the CqNAC family is shown in Figure 2c, and their multilevel consensus amino acid sequences of motifs are listed in Figure S2. Generally, NAC proteins that were clustered in the same subgroups shared similar motif composition. Motifs 3, and 5, which represented the highly conserved Subdomain D, were shared in the CqNAC family. Motifs 2, 4, 1, and 6, corresponding to Subdomains A, B, C, and E, also existed in most CqNACs. Meanwhile, a DNA-binding domain (DBD) existed in Subdomain C, and a nuclear localization signal (NLS) was found in Subdomain D (Figure S3). PSORT and Cello analysis showed that most of the NACs in quinoa are localized to the nucleus (Table S3).

### 3.4. Chromosomal Localization and Duplication of NACs in Quinoa

**Figure 3:**
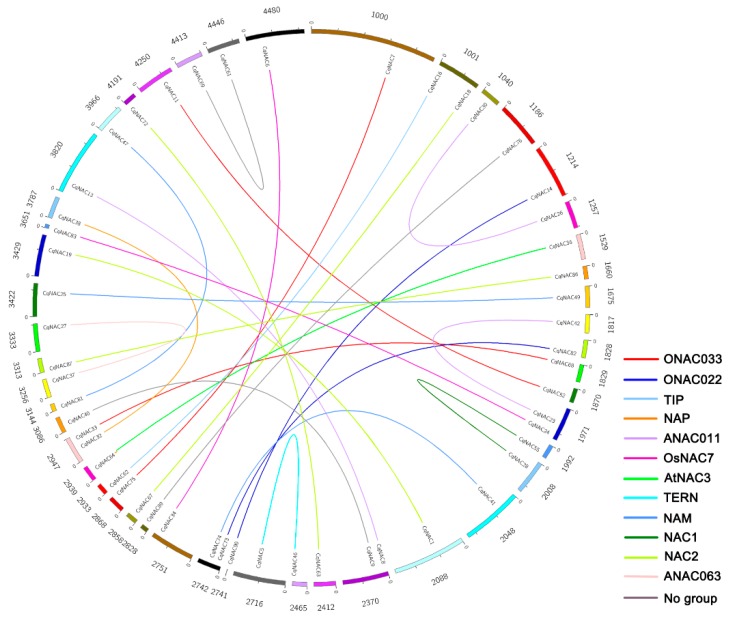
Distribution of 28 duplicated *NAC* gene pairs in quinoa. The numbers represent the scaffolds of quinoa which *CqNACs* are located in. The duplicated gene pairs are joined by colored lines. The differently colored lines represent the subfamilies within the *CqNAC* family.

The genomic localization of *CqNACs* was displayed in Table S4. Gene duplication events were investigated to elucidate the expansion patterns of the *NAC* family in quinoa. In this study, 28 duplicated gene pairs were identified, and these gene pairs were concentrated in NAC2, ANAC011, NAM, and ONAC003 subfamilies (Figure 3, Table 1). In addition, the Ka/Ks ratio of each duplicated gene pair was calculated to assess the molecular evolutionary rates (Table 1). All of Ka/Ks ratios for the duplicated *CqNACs* were less than 1.

### 3.5. Expression Profiles of NACs in Quinoa

**Figure 4:**
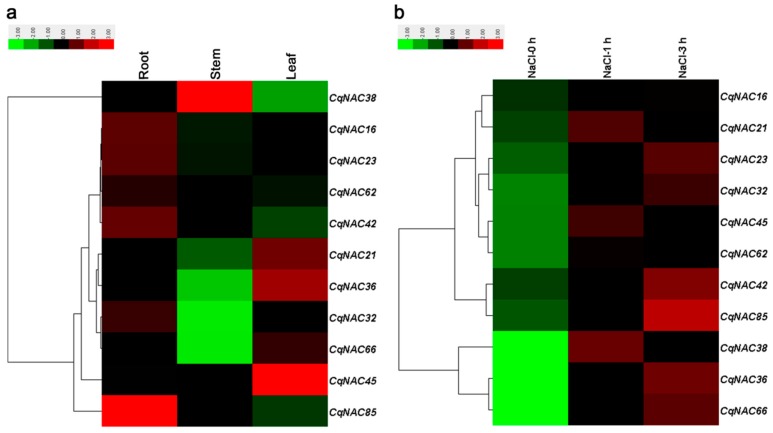
Expression profiles of 11 *CqNAC* genes in different tissues (**a**) and in root under salt stress treatment (**b**). The color bar at the top of the heat map represents relative expression values.

Previous studies have demonstrated that some *NAC* genes in *Arabidopsis*, such as *ANAC019*/AT1G52890 [50], *RD26*/AT4G27410 [50,51], *ANAC2*/AT3G15510 [21], *NAC096*/AT5G46590 [35], *ATAF1*/AT1G01720 [52], *NTL6*/AT3G49530 [53], *NTL8*/AT2G27300 [54], and *ANAC042*/AT2G43000 [55], played a role in plant responses to salt stress, as well as other abiotic stresses. In the current study, 11 *CqNACs* which showed high orthology to these *AtNACs* (Figure 1, Table S6) were selected, and the expression patterns of the 11 selected *CqNACs* were explored. As shown in Figure 4a, the expression patterns of these *CqNACs* varied significantly in different tissues. *CqNAC38* was predominantly expressed in stems, while *CqNAC4*5, *CqNAC36*, *CqNAC21*, and *CqNAC66* expressed at a high level in leaves. Other genes, such as *CqNAC85*, *CqNAC16*, *CqNAC23*, *CqNAC42*, *CqNAC62*, and *CqNAC32*, shared a high expression level in roots. Additionally, to further confirm the salt stress response of *CqNACs*, the expression profiles of 11 *CqNACs* in the roots of seedlings under salt stress were investigated (Figure 4b). The result revealed that the expression of all 11 selected *CqNACs* were upregulated in response to salt stress. Among them, *CqNAC36*, *CqNAC38*, *CqNAC66*, and *CqNAC85* showed a significant increase in expression levels after salt stress treatment. Moreover, the expression patterns of 4 duplicated *CqNAC* gene pairs were compared (Figure S4). Among them, 3 paired genes (*CqNAC16*/*CqNAC62*, *CqNAC23*/*CqNAC42*, and *CqNAC32*/*CqNAC38*) shared almost equivalent expression profiles in the tested tissues and root after salt treatment (Figure S4a–c,e–g), but this was not the case for *CqNAC32*/*CqNAC38*. The expression patterns of the duplicated gene pair were strongly divergent (Figure S4d,h).

## 4. Discussion

With quinoa being a highly stress-tolerant crop, sequencing of its genome facilitates our identification of resistant genes and genetic improvement of crops. The present research performed a genome-wide analysis of the *NAC* genes in the emerging crop quinoa.

In the present study, we identified a total of 90 *NACs* genes in quinoa (Table S1). The *CqNACs* varied substantially in the size, sequence, and the physicochemical properties of their encoded proteins, which were comparable with *NACs* from other plant species [12,31,33]. Through phylogenetic classification, 14 *NAC* subfamilies were clustered in quinoa (Figure 1, Figure 2a, Table S2). Among them, the OsNAC7 subfamily had the most *CqNACs* (14%), followed by the NAC2 (12%) and NAM (11%) subfamilies, whereas the OsNAC8 (1%) and ATAF (1%) subfamilies had the least genes (Figure S1) and a similar distribution of the *NAC* family was found in cassava [33] and cotton [12]. However, not all the subfamilies were present in quinoa. Compared with the *NAC* members in *Arabidopsis*, no *CqNAC* members were identified in ANAC001 and SENU5 subfamilies, suggesting that quinoa might have experienced gene loss in these subfamilies.

To investigate the structural features of *CqNAC* genes, gene structures and protein motifs were analyzed, and the distribution of introns/exons and conserved motifs confirmed the similar phylogenetic classification (Figure 2). In the present study, the number of introns of *CqNACs* varied from 0 to 5 (Figure 2b, Table S2). Most of *CqNACs* contained introns; only 3 of the *CqNACs* were intronless, and the predominant gene structure was three exons and two introns, indicating a similar gene structure diversity as *NACs* in other species [12,33]. The differences in gene structure of *CqNAC* genes result from the gains and losses of introns, and the diverse status of gene structure might be meaningful for *CqNAC* gene evolution and could thus facilitate evolutionary gene co-option for new functions to help plants adapt to environmental changes [12,31,33]. 

In this study, 20 distinct conserved motifs were identified and classified based on sequence similarity of the conserved motifs (Figure 2c, Figure S2). CqNAC proteins clustered in the same subfamilies shared similar motif composition, indicating functional similarities among members of the same subfamilies. Like NAC families in other species, the CqNAC family contained the NAC domain and TAR region, and Subdomains A, C, and D were highly conserved, while Subdomains B and E were relatively divergent (Figure 2c, Figure S3) [10,31]. In addition, a DNA binding domain (DBD) was identified in Subdomain C, which suggested that Subdomain C might be involved in DNA binding [56]. In addition, the putative NLS existed in Subdomain D, and most of the CqNACs were predicted to be nuclear proteins through PSORT and Cello analysis (Table S3). The result has been confirmed for some NACs through subcellular localization [17,23,57].

To elucidate the expanded mechanism of the *NAC* gene family in quinoa, gene duplication events were investigated (Figure 3, Table 1). We identified a total of 28 duplicated *CqNAC* gene pairs. The duplication events showed a strong expansion preference for some *CqNAC* subfamilies, including NAC2, ANAC011, NAM, and ONAC003 subfamilies (Figure 3, Table 1). Thus, the duplication event was not random across *NAC* subfamilies during quinoa evolution. Moreover, all of the Ka/Ks ratios for the duplicated *CqNAC* gene pairs were less than 1, suggesting that the *CqNACs* might have mainly experienced purifying selection (Table 1). As purifying selection apparently constrains the divergence of the duplicated genes, the results indicated that the duplicated *CqNAC* genes might have retained some essential functions during sequent evolution [42,43].

The analysis of gene expression profiles can provide crucial clues for functional assessment. The result in this study indicated the 11 selected *CqNACs* showed significant tissue-specific expression patterns (Figure 4a), suggesting that they might play diverse roles in quinoa plant growth and development. Moreover, NAC proteins are plant-specific TFs that have been shown to function in salt stress responses [21,22,23]. With the goal of identifying candidate salt stress-responsive *CqNAC* genes, we performed the analysis of expression patterns of selected *CqNAC* genes (Figure 4b). In this study, all the 11 *CqNAC* were positively regulated in response to salt stress. Among them, *CqNAC36*, *CqNAC66* (orthologous to *ANAC019* and *RD26*), *CqNAC38* (orthologous to *ANAC2*), and *CqNAC85* (orthologous to *ANAC042*) were significantly upregulated. These genes may play a role in the regulation of transcriptional reprogramming associated with plant responses to salt stress and contribute to the establishment of complex signaling networks in plant [58]. They can be selected as candidate genes for further characterization of their functional involvement in plant resistance to salt stress and provide an opportunity to further understand the mechanisms involved in quinoa’s strong salinity tolerance. Moreover, the expression profiles of duplicated *CqNAC* genes showed that 3 pairs of duplicated genes shared similar expression patterns (Figure S4). The result suggested that these duplicated genes might retain some essential functions during subsequent evolution, and the similar expression profiles might be related to their highly similar protein architecture, together with *cis*-regulatory elements [12,31]. However, the duplicated genes *CqNAC32*/*CqNAC38* showed significant expression divergence, which might be caused by variation in gene regulation after the duplication events, and the differential expression patterns of duplicated *CqNAC* genes demonstrated that they might have experienced functionalization during the evolutionary process [31,59].

In conclusion, we identified a total of 90 *NAC* genes in the quinoa genome and investigated the important features of the gene family, such as basic classification, expansion patterns, and expression profiles. These findings lay an important foundation for identification and molecular evolution of the *NAC* family in quinoa and provide gene resources for the functional characterization of *CqNAC* genes involved in salinity tolerance. 

## Figures and Tables

**Table 1:** Ka/Ks analysis for duplicated gene pairs of *NACs* in quinoa.

Duplicated gene 1	Duplicated gene 2	Subfamily	Ka	Ks	Ka/Ks	Purifing Selection
*CqNAC1*	*CqNAC19*	NAC2	0.0599	0.1309	0.4576	Yes
*CqNAC2*	*CqNAC20*	NAC2	0.0594	0.0732	0.8115	Yes
*CqNAC5*	*CqNAC46*	TERN	0.0275	0.0913	0.3012	Yes
*CqNAC6*	*CqNAC34*	OsNAC7	0.0069	0.1997	0.0346	Yes
*CqNAC7*	*CqNAC75*	ONAC003	0.0133	0.0743	0.1790	Yes
*CqNAC8*	*CqNAC13*	ANAC011	0.0167	0.1202	0.1389	Yes
*CqNAC9*	*CqNAC40*	No group	0.0256	0.1162	0.2203	Yes
*CqNAC11*	*CqNAC52*	ONAC003	0.0347	0.1097	0.3163	Yes
*CqNAC14*	*CqNAC90*	ONAC022	0.0300	0.1093	0.2745	Yes
*CqNAC16*	*CqNAC62*	TIP	0.0206	0.0690	0.2986	Yes
*CqNAC18*	*CqNAC67*	NAC2	0.0289	0.0816	0.3542	Yes
*CqNAC23*	*CqNAC42*	ANAC011	0.0161	0.0520	0.3096	Yes
*CqNAC24*	*CqNAC83*	OsNAC7	0.0093	0.1146	0.0812	Yes
*CqNAC25*	*CqNAC49*	NAM	0.0084	0.1470	0.0571	Yes
*CqNAC26*	*CqNAC30*	ANAC011	0.0061	0.1014	0.0602	Yes
*CqNAC27*	*CqNAC37*	ANAC063	0.0299	0.0434	0.6889	Yes
*CqNAC28*	*CqNAC55*	NAC1	0.0000	0.0749	0.0000	Yes
*CqNAC32*	*CqNAC38*	NAP	0.0177	0.1183	0.1496	Yes
*CqNAC33*	*CqNAC68*	ONAC003	0.0175	0.1265	0.1383	Yes
*CqNAC36*	*CqNAC66*	AtNAC3	0.2210	0.4130	0.5351	Yes
*CqNAC41*	*CqNAC74*	NAM	0.0136	0.0922	0.1475	Yes
*CqNAC47*	*CqNAC81*	NAM	0.0119	0.0813	0.1464	Yes
*CqNAC61*	*CqNAC69*	No group	0.0164	0.0598	0.2742	Yes
*CqNAC63*	*CqNAC72*	NAC2	0.0249	0.1268	0.1964	Yes
*CqNAC64*	*CqNAC65*	NAP	0.0474	0.0732	0.6475	Yes
*CqNAC73*	*CqNAC82*	ONAC022	0.0158	0.1028	0.1537	Yes
*CqNAC76*	*CqNAC89*	No group	0.0085	0.0901	0.0943	Yes
*CqNAC86*	*CqNAC87*	NAC2	0.0152	0.0888	0.1712	Yes

## Supplementary Materials

The following are available online at https://www.mdpi.com/2073-4425/10/7/500/s1, Figure S1: The percentage of members in each *NAC* subfamily in quinoa. Figure S2: Multilevel consensus sequence and their logo of CqNAC proteins as predicted by MEME web server. Figure S3: Conserved domain analysis of the CqNACs using the WebLogo program. Figure S4: Expression patterns of some duplicated *CqNAC* genes in different organs (a–d) and in root under salt stress treatment (e–h). Table S1: The structural analysis of NACs identified in this study. Table S2: The classification and gene structures of *NACs* in quinoa. Table S3: Subcellular localization of NAC proteins in quinoa. Table S4: Genomic location of *NACs* in quinoa. Table S5: PCR primers used for qRT-PCR in this study. Table S6: Orthology information of CqNACs and AtNACs from BLASTP.
