# Supplementary material for: Genome-Wide Identification, Characterization, and Expression Analysis of the NAC Transcription Factor in Chenopodium quinoa
Source: Genes (Basel). 2019 Jun 30;10(7):500. doi: 10.3390/genes10070500 (PMC6678211; doi:10.3390/genes10070500)
Supplement: Supplementary file 1 [file genes-10-00500-s001.pdf]

Electronic Supplementary Information

Genome-wide identification, characterization and expression analysis of the NAC transcription factor in *Chenopodium quinoa*

Feng Li[1,2], Xuhu Guo[1,2], Jianxia Liu[1,2], Feng Zhou[1,2], Wenying Liu[1,2], Juan Wu[1,2], Hongli Zhang[1,2], Huifen Cao[1,2], Huanzhen Su[1,2], Riyu Wen[3*]

1 College of Life Science, Shanxi Datong University, Datong 037009, People's Republic of China
2 Research and Development Center of Agricultural Facility Technology, Shanxi Datong University, Datong 037009, People's Republic of China
3 Maize Research Institute, Shanxi Academy of Agricultural Sciences, Xinzhou 034000, People's Republic of China
* Corresponding author: Riyu Wen. Email: lingchenhaifeng@163.com. Telephone number: 086-0350-8622619.

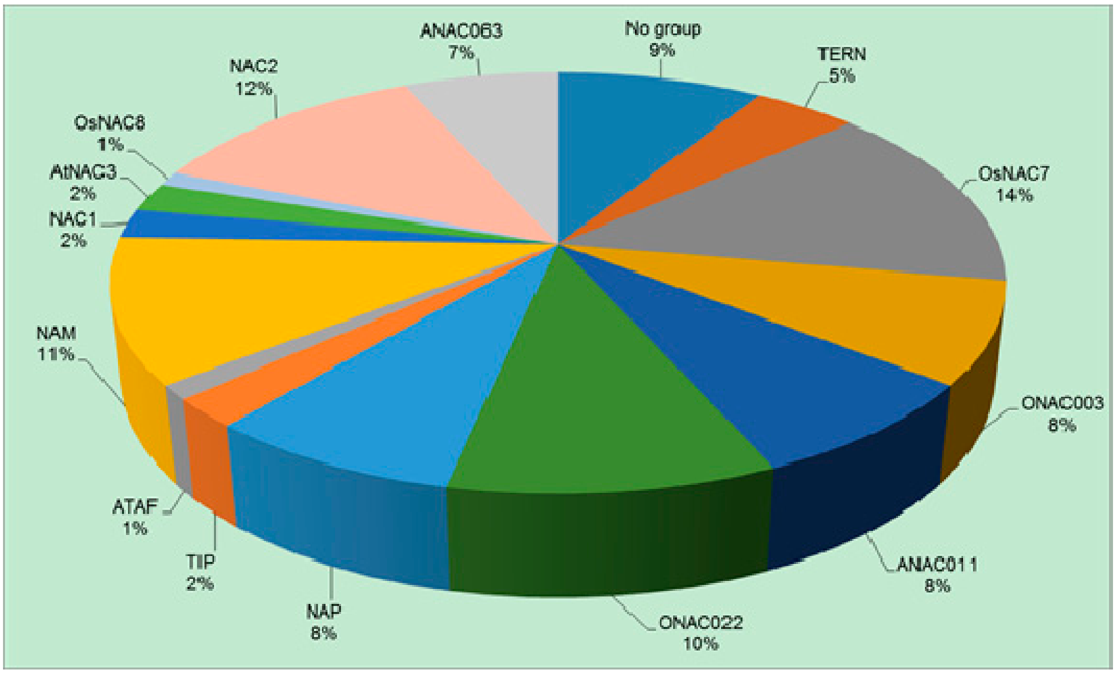

Figure S1. The percentage of members in each *NAC* subfamily in quinoa.

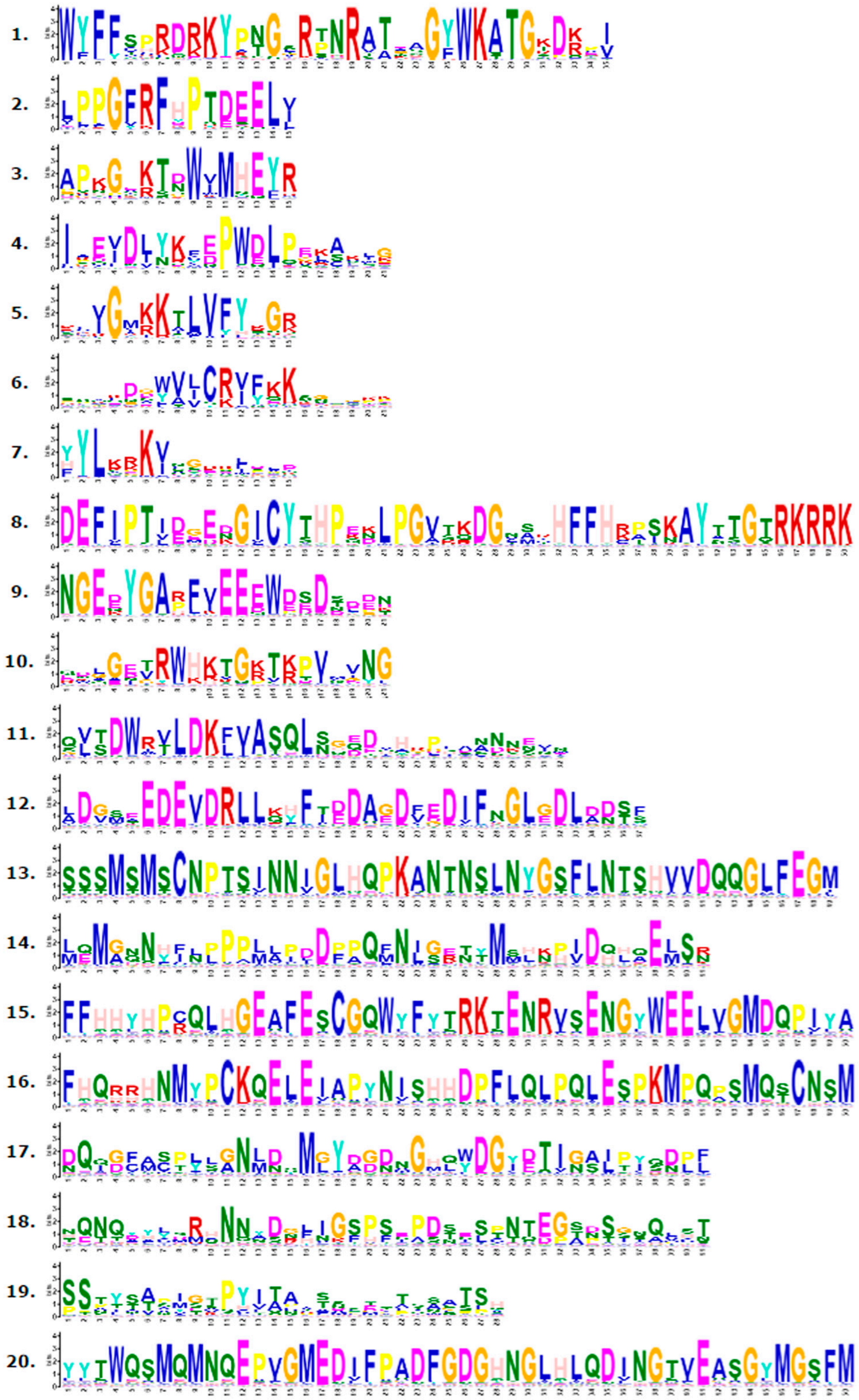

Figure S2. Multilevel consensus sequence and their logo of CqNAC proteins as predicted by MEME web server.

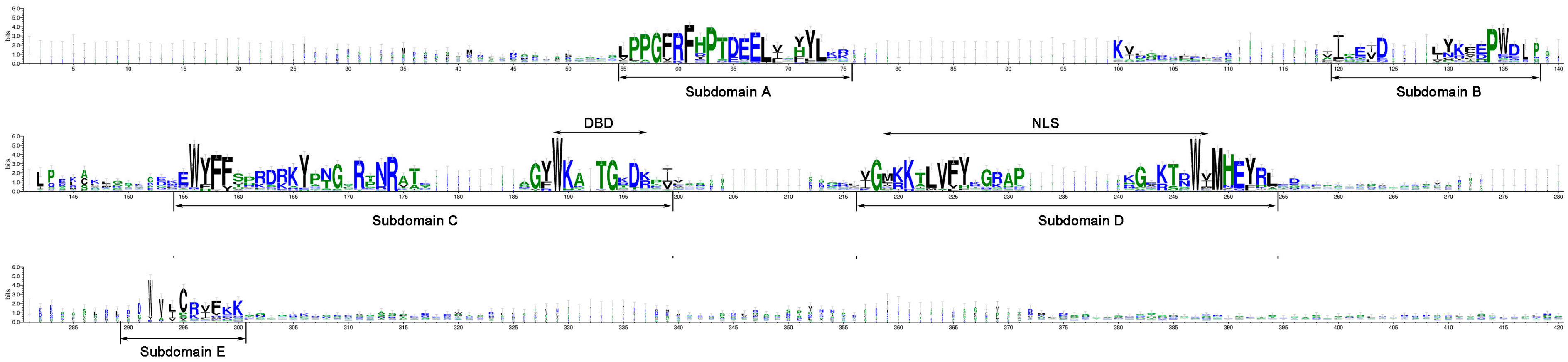

Figure S3. Conserved domain analysis of the CqNACs using the WebLogo program. The height of the letter designating the amino acid residue at each position represents the degree of conservation. The numbers on the x-axis represent the sequence positions in the corresponding conserved domains. The y-axis represents the information content measured in bits.

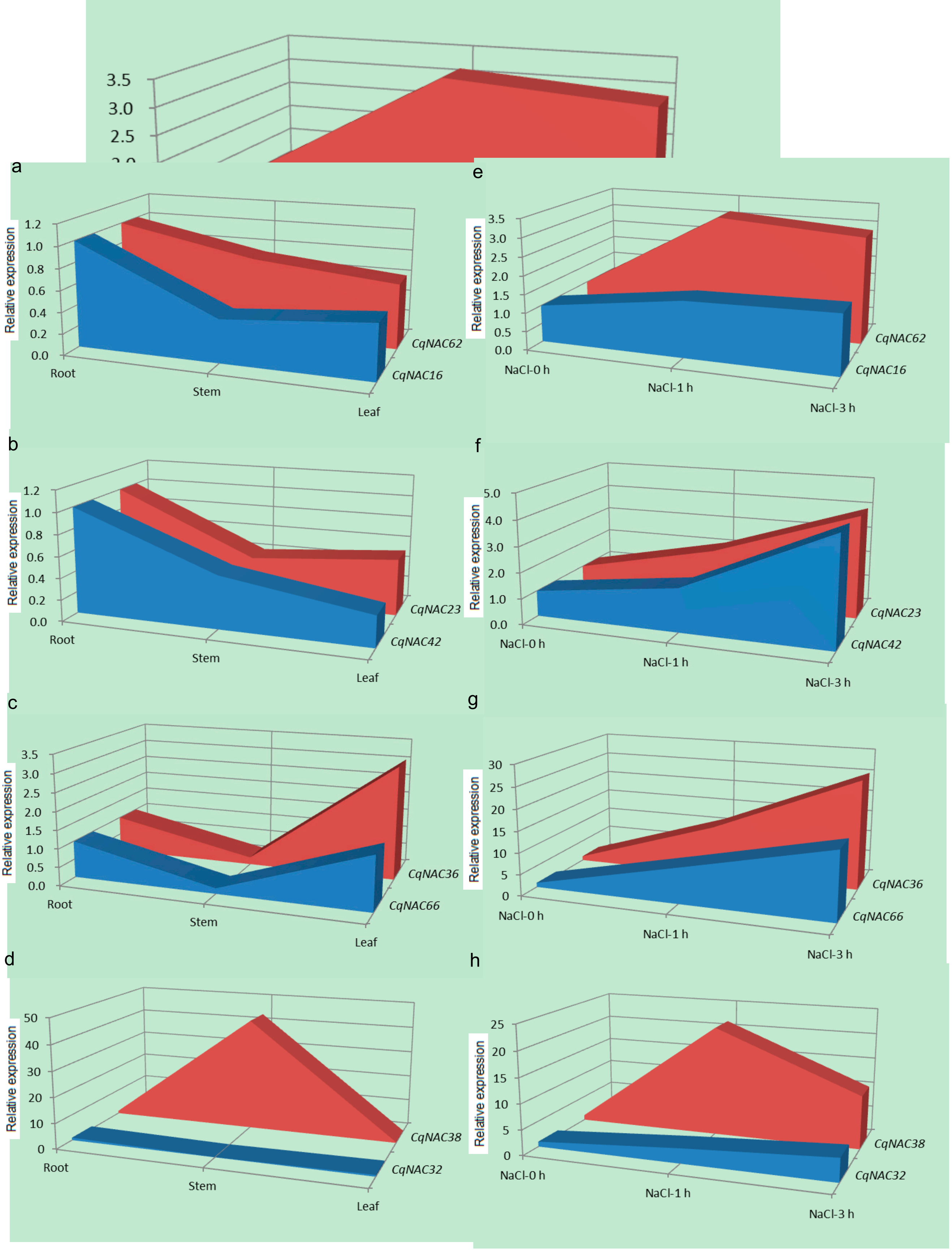

Figure S4. Expression patterns of some duplicated *CqNAC* genes in different organs (**a**–**d**) and in root under salt stress treatment (**e**–**h**).

Table 1. The structural analysis of CqNACs identified in this study.

Name	Locus name	Protein length (AA)	α-helix	Extended strand	β-turn	Random coil	Molecular weight	Theoretical pI
CqNAC1	AUR62000492-RA	327	28.75%	12.84%	4.89%	53.52%	36465.01	5.21
CqNAC2	AUR62000493-RA	329	28.27%	11.85%	4.26%	55.62%	36054.67	5.15
CqNAC3	AUR62000898-RA	418	12.68%	29.19%	8.85%	49.28%	46498.08	6.47
CqNAC4	AUR62001365-RA	307	34.20%	15.64%	3.26%	46.91%	35908.02	5.42
CqNAC5	AUR62001522-RA	268	21.64%	16.79%	3.73%	57.84%	30289.73	8.74
CqNAC6	AUR62002191-RA	249	19.28%	17.67%	6.02%	57.03%	28433.49	6.07
CqNAC7	AUR62002922-RA	339	22.12%	17.70%	5.31%	54.87%	38180.40	4.88
CqNAC8	AUR62003400-RA	573	27.92%	11.52%	4.01%	56.54%	65093.66	5.67
CqNAC9	AUR62003583-RA	224	27.23%	16.07%	5.80%	50.89%	26274.26	5.27
CqNAC10	AUR62004554-RA	334	24.25%	13.47%	5.69%	56.59%	38417.87	6.47
CqNAC11	AUR62004572-RA	388	24.23%	15.98%	5.41%	54.38%	42975.83	5.18
CqNAC12	AUR62004648-RA	334	20.66%	15.27%	4.19%	59.88%	38928.20	6.34
CqNAC13	AUR62004778-RA	576	27.08%	11.98%	4.34%	56.60%	65290.07	5.84
CqNAC14	AUR62005772-RA	446	23.77%	12.11%	6.95%	57.17%	50303.27	5.27
CqNAC15	AUR62005821-RA	288	35.76%	11.11%	4.17%	48.96%	32911.82	5.52
CqNAC16	AUR62005966-RA	631	26.31%	17.12%	5.71%	50.87%	70214.52	5.11
CqNAC17	AUR62006223-RA	276	15.22%	18.84%	3.62%	62.32%	31807.15	8.44
CqNAC18	AUR62006407-RA	372	25.27%	13.17%	3.49%	58.06%	40436.27	4.64
CqNAC19	AUR62006836-RA	315	34.29%	15.87%	4.13%	45.71%	34978.40	4.79
CqNAC20	AUR62006837-RA	319	22.57%	13.79%	5.02%	58.62%	35107.58	4.97
CqNAC21	AUR62006885-RA	329	20.06%	11.25%	5.17%	63.53%	37188.40	8.05

CqNAC22	AUR62007071-RA	279	20.07%	14.70%	5.38%	59.86%	32176.90	6.08
CqNAC23	AUR62007247-RA	327	10.40%	12.23%	3.98%	73.39%	37313.61	5.03
CqNAC24	AUR62007469-RA	370	20.27%	14.32%	2.97%	62.43%	42954.10	6.34
CqNAC25	AUR62008438-RA	364	20.33%	11.26%	4.40%	64.01%	40914.70	6.32
CqNAC26	AUR62010080-RA	352	23.58%	9.66%	4.26%	62.50%	40279.01	5.35
CqNAC27	AUR62011638-RA	414	20.53%	18.12%	11.59%	49.76%	46988.95	4.89
CqNAC28	AUR62011853-RA	145	16.55%	25.52%	9.66%	48.28%	16945.51	9.25
CqNAC29	AUR62012948-RA	146	10.27%	23.97%	11.64%	54.11%	17407.86	9.61
CqNAC30	AUR62013074-RA	352	21.59%	9.66%	5.11%	63.64%	40140.84	5.35
CqNAC31	AUR62013541-RA	346	24.57%	13.01%	3.47%	58.96%	**	**
CqNAC32	AUR62014768-RA	343	9.91%	18.37%	3.50%	68.22%	38527.15	8.34
CqNAC33	AUR62014879-RA	252	9.52%	22.22%	8.33%	59.92%	28592.20	8.35
CqNAC34	AUR62015520-RA	248	19.76%	20.16%	4.44%	55.65%	28310.33	5.91
CqNAC35	AUR62015629-RA	302	20.20%	17.22%	3.97%	58.61%	33805.23	9.02
CqNAC36	AUR62015630-RA	278	22.66%	15.11%	3.96%	58.27%	30847.92	8.97
CqNAC37	AUR62016213-RA	389	20.57%	17.48%	10.28%	51.67%	44124.93	5.19
CqNAC38	AUR62016954-RA	327	15.29%	18.65%	2.14%	63.91%	36484.83	8.68
CqNAC39	AUR62017681-RA	418	16.27%	18.90%	4.55%	60.29%	46777.01	8.28
CqNAC40	AUR62017896-RA	224	25.45%	14.73%	4.91%	54.91%	26482.56	5.10
CqNAC41	AUR62018102-RA	333	12.91%	14.71%	3.60%	68.77%	38348.33	5.25
CqNAC42	AUR62018752-RA	325	9.85%	12.62%	3.08%	74.46%	37194.42	5.41
CqNAC43	AUR62019477-RA	400	15.25%	25.75%	7.50%	51.50%	**	**
CqNAC44	AUR62019936-RA	341	24.05%	12.02%	4.11%	59.82%	39174.79	6.72
CqNAC45	AUR62020251-RA	430	28.37%	12.09%	5.58%	53.95%	48339.23	9.30

CqNAC46	AUR62020261-RA	258	20.54%	12.40%	3.10%	63.95%	29053.47	8.57
CqNAC47	AUR62020634-RA	297	18.52%	14.14%	3.37%	63.97%	33732.06	8.90
CqNAC48	AUR62021621-RA	198	27.27%	20.20%	7.58%	44.95%	22824.18	9.30
CqNAC49	AUR62021652-RA	361	18.84%	13.02%	4.16%	63.99%	40521.21	6.32
CqNAC50	AUR62022486-RA	386	32.64%	16.32%	4.40%	46.63%	43144.30	7.13
CqNAC51	AUR62022669-RA	250	20.00%	19.20%	7.60%	53.20%	28785.28	8.67
CqNAC52	AUR62022685-RA	374	18.72%	17.11%	5.08%	59.09%	41416.33	5.17
CqNAC53	AUR62022690-RA	571	25.04%	19.79%	6.13%	49.04%	64944.45	6.49
CqNAC54	AUR62024101-RA	248	25.04%	21.37%	8.47%	44.76%	28076.95	9.10
CqNAC55	AUR62024698-RA	149	16.11%	24.16%	8.72%	51.01%	17465.06	9.06
CqNAC56	AUR62025580-RA	342	12.87%	25.15%	9.36%	52.63%	39460.28	5.56
CqNAC57	AUR62025636-RA	354	30.79%	17.23%	5.08%	46.89%	41347.03	5.22
CqNAC58	AUR62026741-RA	283	19.79%	18.37%	4.95%	56.89%	32508.34	6.90
CqNAC59	AUR62026904-RA	344	16.86%	15.70%	4.07%	63.37%	39416.95	6.46
CqNAC60	AUR62027152-RA	222	17.57%	22.97%	4.95%	54.50%	25666.26	8.37
CqNAC61	AUR62027390-RA	168	17.26%	21.43%	5.95%	55.36%	19591.24	7.02
CqNAC62	AUR62028103-RA	630	28.41%	13.17%	2.86%	55.56%	70372.71	5.19
CqNAC63	AUR62029057-RA	524	27.67%	11.45%	3.05%	57.82%	58769.41	4.96
CqNAC64	AUR62029330-RA	328	17.99%	16.77%	4.27%	60.98%	36617.18	8.80
CqNAC65	AUR62029331-RA	327	19.57%	17.13%	2.45%	60.86%	36697.29	8.23
CqNAC66	AUR62029344-RA	298	17.45%	17.45%	4.70%	60.40%	33304.44	9.31
CqNAC67	AUR62029755-RA	368	21.74%	14.13%	4.35%	59.78%	40136.91	4.66
CqNAC68	AUR62031086-RA	251	13.55%	19.52%	6.37%	60.56%	28513.14	7.92
CqNAC69	AUR62031305-RA	158	18.99%	13.92%	3.80%	63.29%	18487.95	7.06

CqNAC70	AUR62032816-RA	272	23.16%	16.18%	3.31%	57.35%	30891.77	8.65
CqNAC71	AUR62033276-RA	319	27.59%	16.30%	4.70%	51.41%	35952.06	6.83
CqNAC72	AUR62033952-RA	530	29.62%	12.08%	3.02%	55.28%	59469.49	5.05
CqNAC73	AUR62034400-RA	385	20.00%	8.05%	3.38%	68.57%	43291.89	8.27
CqNAC74	AUR62034435-RA	341	15.54%	14.37%	3.52%	66.57%	39116.19	5.19
CqNAC75	AUR62035283-RA	365	24.66%	16.44%	4.93%	53.97%	4101.73	5.02
CqNAC76	AUR62035514-RA	153	16.34%	24.84%	5.23%	53.59%	17543.23	9.51
CqNAC77	AUR62036158-RA	324	16.05%	19.75%	5.25%	58.95%	35672.96	8.15
CqNAC78	AUR62036626-RA	279	24.73%	18.28%	3.94%	53.05%	31455.38	6.51
CqNAC79	AUR62036991-RA	151	14.57%	20.53%	4.64%	60.26%	17509.63	8.64
CqNAC80	AUR62037478-RA	241	16.18%	20.75%	7.05%	56.02%	27617.77	5.43
CqNAC81	AUR62038748-RA	294	14.29%	10.88%	3.74%	71.09%	33605.95	8.75
CqNAC82	AUR62039815-RA	385	20.26%	6.23%	3.38%	70.13%	43123.80	8.28
CqNAC83	AUR62039927-RA	370	25.41%	13.24%	2.43%	58.92%	42952.16	6.24
CqNAC84	AUR62040120-RA	390	24.10%	15.13%	7.44%	53.33%	43352.17	9.15
CqNAC85	AUR62040889-RA	213	26.76%	20.19%	5.16%	47.89%	24342.91	9.15
CqNAC86	AUR62041525-RA	550	20.91%	17.27%	5.64%	56.18%	61100.19	4.60
CqNAC87	AUR62041539-RA	556	23.20%	16.37%	5.40%	55.04%	61749.78	4.60
CqNAC88	AUR62043491-RA	393	29.52%	9.67%	5.09%	55.73%	43868.48	5.37
CqNAC89	AUR62043497-RA	155	10.32%	29.68%	4.52%	55.48%	17813.61	9.51
CqNAC90	AUR62044373-RA	454	25.77%	11.23%	3.96%	59.03%	51258.61	5.15

Table S2: The classification and gene structures of *NACs* in quinoa.

Name	Subfamily	Extron number	Intron number
CqNAC1	NAC2	2	1
CqNAC2	NAC2	1	0
CqNAC3	ANAC063	3	2
CqNAC4	ANAC063	3	2
CqNAC5	TERN	4	3
CqNAC6	OsNAC7	4	3
CqNAC7	ONAC003	5	4
CqNAC8	ANAC011	6	5
CqNAC9	No group	3	2
CqNAC10	OsNAC7	2	1
CqNAC11	ONAC003	5	4
CqNAC12	OsNAC7	3	2
CqNAC13	ANAC011	6	5
CqNAC14	ONAC022	3	2
CqNAC15	NAP	5	4
CqNAC16	TIP	5	4
CqNAC17	TERN	3	2
CqNAC18	NAC2	1	0
CqNAC19	NAC2	2	1
CqNAC20	NAC2	2	1
CqNAC21	ATAF	4	3
CqNAC22	OsNAC7	3	2
CqNAC23	ANAC011	4	3
CqNAC24	OsNAC7	3	2
CqNAC25	NAM	3	2
CqNAC26	ANAC011	4	3
CqNAC27	ANAC063	3	2
CqNAC28	NAC1	2	1
CqNAC29	ONAC022	2	1
CqNAC30	ANAC011	4	3
CqNAC31	OsNAC7	3	2
CqNAC32	NAP	3	2
CqNAC33	ONAC003	2	1
CqNAC34	OsNAC7	4	3
CqNAC35	NAP	3	2
CqNAC36	AtNAC3	4	3
CqNAC37	ANAC063	3	2

CqNAC38	NAP	3	2
CqNAC39	OsNAC7	4	3
CqNAC40	No group	3	2
CqNAC41	NAM	3	2
CqNAC42	ANAC011	4	3
CqNAC43	NAM	3	2
CqNAC44	OsNAC7	3	2
CqNAC45	OsNAC8	5	4
CqNAC46	TERN	3	2
CqNAC47	NAM	4	3
CqNAC48	NAM	4	3
CqNAC49	NAM	3	2
CqNAC50	No group	6	5
CqNAC51	OsNAC7	3	2
CqNAC52	ONAC003	5	4
CqNAC53	OsNAC7	5	4
CqNAC54	NAM	4	3
CqNAC55	NAC1	3	2
CqNAC56	ANAC063	3	2
CqNAC57	ANAC063	3	2
CqNAC58	OsNAC7	4	3
CqNAC59	ONAC022	3	2
CqNAC60	ONAC022	3	2
CqNAC61	No group	2	1
CqNAC62	TIP	5	4
CqNAC63	NAC2	4	3
CqNAC64	NAP	4	3
CqNAC65	NAP	4	3
CqNAC66	AtNAC3	4	3
CqNAC67	NAC2	1	0
CqNAC68	ONAC003	2	1
CqNAC69	No group	2	1
CqNAC70	NAP	3	2
CqNAC71	ONAC003	5	4
CqNAC72	NAC2	5	4
CqNAC73	ONAC022	3	2
CqNAC74	NAM	3	2
CqNAC75	ONAC003	6	5
CqNAC76	No group	2	1
CqNAC77	NAM	5	4

CqNAC78	No group	3	2
CqNAC79	TERN	2	1
CqNAC80	ANAC011	3	2
CqNAC81	NAM	4	3
CqNAC82	ONAC022	3	2
CqNAC83	OsNAC7	3	2
CqNAC84	ONAC022	5	4
CqNAC85	ONAC022	3	2
CqNAC86	NAC2	6	5
CqNAC87	NAC2	6	5
CqNAC88	NAC2	4	3
CqNAC89	No group	2	1
CqNAC90	ONAC022	3	2

Table S3: Subcellular localization of NAC proteins in quinoa.

Name	PSORT result	Cello result
CqNAC1	Nuclear protein	Nuclear protein
CqNAC2	Nuclear protein	Chloroplast protein
CqNAC3	Nuclear protein	Nuclear protein
CqNAC4	Nuclear protein	Nuclear protein
CqNAC5	Nuclear protein	Nuclear protein
CqNAC6	Peroxisomal protein	Nuclear protein
CqNAC7	Nuclear protein	Nuclear protein
CqNAC8	Nuclear protein	Nuclear protein
CqNAC9	Nuclear protein	Nuclear protein
CqNAC10	Nuclear protein	Nuclear protein
CqNAC11	Nuclear protein	Nuclear protein
CqNAC12	Nuclear protein	Nuclear protein
CqNAC13	Nuclear protein	Nuclear protein
CqNAC14	Nuclear protein	Nuclear protein
CqNAC15	Nuclear protein	Nuclear protein
CqNAC16	Nuclear protein	Nuclear protein
CqNAC17	Nuclear protein	Nuclear protein
CqNAC18	Nuclear protein	Nuclear protein
CqNAC19	Nuclear protein	Nuclear protein
CqNAC20	Nuclear protein	Nuclear protein
CqNAC21	Chloroplast protein	Nuclear protein
CqNAC22	Peroxisomal protein	Nuclear protein
CqNAC23	Nuclear protein	Nuclear protein
CqNAC24	Nuclear protein	Nuclear protein
CqNAC25	Nuclear protein	Nuclear protein
CqNAC26	Chloroplast protein	Nuclear protein
CqNAC27	Nuclear protein	Nuclear protein
CqNAC28	Peroxisomal protein	Cytoplasmic protein
CqNAC29	Nuclear protein	Cytoplasmic protein
CqNAC30	Nuclear protein	Nuclear protein
CqNAC31	Peroxisomal protein	Nuclear protein
CqNAC32	Nuclear protein	Nuclear protein
CqNAC33	Nuclear protein	Nuclear protein
CqNAC34	Peroxisomal protein	Nuclear protein
CqNAC35	Nuclear protein	Nuclear protein
CqNAC36	Nuclear protein	Nuclear protein
CqNAC37	Nuclear protein	Nuclear protein
CqNAC38	Nuclear protein	Nuclear protein
CqNAC39	Nuclear protein	Nuclear protein
CqNAC40	Nuclear protein	Nuclear protein

CqNAC41	Nuclear protein	Cytoplasmic protein
CqNAC42	Nuclear protein	Nuclear protein
CqNAC43	Nuclear protein	Nuclear protein
CqNAC44	Nuclear protein	Nuclear protein
CqNAC45	Nuclear protein	Nuclear protein
CqNAC46	Peroxisomal protein	Nuclear protein
CqNAC47	Nuclear protein	Nuclear protein
CqNAC48	Cytoplasmic protein	Cytoplasmic protein
CqNAC49	Nuclear protein	Nuclear protein
CqNAC50	Nuclear protein	PlasmaMembrane protein
CqNAC51	Nuclear protein	Nuclear protein
CqNAC52	Nuclear protein	Nuclear protein
CqNAC53	Extracellular protein	Nuclear protein
CqNAC54	Nuclear protein	Cytoplasmic protein
CqNAC55	Peroxisomal protein	Cytoplasmic protein
CqNAC56	Cytoplasmic protein	Nuclear protein
CqNAC57	Nuclear protein	Nuclear protein
CqNAC58	Peroxisomal protein	Nuclear protein
CqNAC59	Nuclear protein	Nuclear protein
CqNAC60	Cytoplasmic protein	Nuclear protein
CqNAC61	Nuclear protein	Nuclear protein
CqNAC62	Cytoplasmic protein	Nuclear protein
CqNAC63	Goloi protein	Nuclear protein
CqNAC64	Nuclear protein	Nuclear protein
CqNAC65	Nuclear protein	Nuclear protein
CqNAC66	Nuclear protein	Nuclear protein
CqNAC67	Cytoplasmic protein	Chloroplast protein
CqNAC68	Nuclear protein	Nuclear protein
CqNAC69	Nuclear protein	Nuclear protein
CqNAC70	Nuclear protein	Nuclear protein
CqNAC71	Nuclear protein	Nuclear protein
CqNAC72	Goloi protein	Nuclear protein
CqNAC73	Nuclear protein	Nuclear protein
CqNAC74	Nuclear protein	Cytoplasmic protein
CqNAC75	Nuclear protein	Nuclear protein
CqNAC76	Nuclear protein	Cytoplasmic protein
CqNAC77	Nuclear protein	Nuclear protein
CqNAC78	Chloroplast protein	Nuclear protein
CqNAC79	Nuclear protein	Nuclear protein
CqNAC80	Nuclear protein	Nuclear protein
CqNAC81	Nuclear protein	Nuclear protein
CqNAC82	Nuclear protein	Nuclear protein

CqNAC83	Nuclear protein	Nuclear protein
CqNAC84	Nuclear protein	Nuclear protein
CqNAC85	Cytoplasmic protein	Nuclear protein
CqNAC86	Chloroplast protein	Cytoplasmic protein
CqNAC87	Chloroplast protein	Vacuole protein
CqNAC88	Chloroplast protein	Nuclear protein
CqNAC89	Nuclear protein	Cytoplasmic protein
CqNAC90	Nuclear protein	Nuclear protein

Table S4: Genomic location of *NACs* in quinoa.

Name	Chromosome Name	Gene Start (bp)	Gene End (bp)
CqNAC1	C_Quinoa_Scaffold_2088	5104189	5105235
CqNAC2	C_Quinoa_Scaffold_2088	5126097	5127086
CqNAC3	C_Quinoa_Scaffold_2088	10436292	10439135
CqNAC4	C_Quinoa_Scaffold_2716	3667151	3670126
CqNAC5	C_Quinoa_Scaffold_2716	5750306	5754005
CqNAC6	C_Quinoa_Scaffold_4480	3338123	3340057
CqNAC7	C_Quinoa_Scaffold_1000	16136114	16139389
CqNAC8	C_Quinoa_Scaffold_2370	206197	210326
CqNAC9	C_Quinoa_Scaffold_2370	2346420	2348358
CqNAC10	C_Quinoa_Scaffold_4250	4675233	4677254
CqNAC11	C_Quinoa_Scaffold_4250	4929761	4932822
CqNAC12	C_Quinoa_Scaffold_4250	5835845	5837591
CqNAC13	C_Quinoa_Scaffold_3820	2201216	2205271
CqNAC14	C_Quinoa_Scaffold_1214	7730160	7733025
CqNAC15	C_Quinoa_Scaffold_1214	8710235	8716062
CqNAC16	C_Quinoa_Scaffold_1001	780823	785367
CqNAC17	C_Quinoa_Scaffold_1001	4184394	4188756
CqNAC18	C_Quinoa_Scaffold_1001	7434695	7435813
CqNAC19	C_Quinoa_Scaffold_3429	5501991	5503001
CqNAC20	C_Quinoa_Scaffold_3429	5506671	5507714
CqNAC21	C_Quinoa_Scaffold_3429	6081924	6086002
CqNAC22	C_Quinoa_Scaffold_1971	870150	871578
CqNAC23	C_Quinoa_Scaffold_1971	3145691	3147716
CqNAC24	C_Quinoa_Scaffold_1971	5582799	5584443
CqNAC25	C_Quinoa_Scaffold_3422	4681091	4683124
CqNAC26	C_Quinoa_Scaffold_1257	1424158	1428569
CqNAC27	C_Quinoa_Scaffold_3333	4561449	4562989
CqNAC28	C_Quinoa_Scaffold_2008	1858490	1862276
CqNAC29	C_Quinoa_Scaffold_1040	720134	720757
CqNAC30	C_Quinoa_Scaffold_1040	1973115	1977583
CqNAC31	C_Quinoa_Scaffold_1611	4650646	4653648
CqNAC32	C_Quinoa_Scaffold_2947	2498542	2500028
CqNAC33	C_Quinoa_Scaffold_2947	4419915	4428541
CqNAC34	C_Quinoa_Scaffold_2751	7542231	7544163
CqNAC35	C_Quinoa_Scaffold_1529	902046	903275
CqNAC36	C_Quinoa_Scaffold_1529	916768	917919
CqNAC37	C_Quinoa_Scaffold_3256	2959547	2961121
CqNAC38	C_Quinoa_Scaffold_3787	1019205	1020764

CqNAC39	C_Quinoa_Scaffold_3458	2937169	2943353
CqNAC40	C_Quinoa_Scaffold_3086	1470978	1472880
CqNAC41	C_Quinoa_Scaffold_2048	3674508	3675854
CqNAC42	C_Quinoa_Scaffold_1817	1668340	1670371
CqNAC43	C_Quinoa_Scaffold_2127	1427472	1429718
CqNAC44	C_Quinoa_Scaffold_1480	2644016	2645684
CqNAC45	C_Quinoa_Scaffold_2465	2584216	2586669
CqNAC46	C_Quinoa_Scaffold_2465	2723997	2725875
CqNAC47	C_Quinoa_Scaffold_3966	1391082	1397450
CqNAC48	C_Quinoa_Scaffold_1675	1474377	1481074
CqNAC49	C_Quinoa_Scaffold_1675	1933365	1935019
CqNAC50	C_Quinoa_Scaffold_3163	7127974	7137645
CqNAC51	C_Quinoa_Scaffold_1870	1636666	1638400
CqNAC52	C_Quinoa_Scaffold_1870	1836151	1839245
CqNAC53	C_Quinoa_Scaffold_1870	1883018	1891940
CqNAC54	C_Quinoa_Scaffold_4119	171296	172432
CqNAC55	C_Quinoa_Scaffold_1992	1842893	1846940
CqNAC56	C_Quinoa_Scaffold_1677	29928	31688
CqNAC57	C_Quinoa_Scaffold_1677	723492	725417
CqNAC58	C_Quinoa_Scaffold_2081	4392587	4400244
CqNAC59	C_Quinoa_Scaffold_2185	2906263	2914672
CqNAC60	C_Quinoa_Scaffold_2314	1809369	1810776
CqNAC61	C_Quinoa_Scaffold_4446	2447430	2449183
CqNAC62	C_Quinoa_Scaffold_2933	546240	550841
CqNAC63	C_Quinoa_Scaffold_2412	3029866	3035495
CqNAC64	C_Quinoa_Scaffold_2939	1713207	1715584
CqNAC65	C_Quinoa_Scaffold_2939	1736295	1738669
CqNAC66	C_Quinoa_Scaffold_2939	1941693	1943007
CqNAC67	C_Quinoa_Scaffold_2858	1227921	1229027
CqNAC68	C_Quinoa_Scaffold_1829	406399	409549
CqNAC69	C_Quinoa_Scaffold_4413	2894460	2896172
CqNAC70	C_Quinoa_Scaffold_3751	595767	597199
CqNAC71	C_Quinoa_Scaffold_2896	1183564	1190468
CqNAC72	C_Quinoa_Scaffold_4191	561057	566172
CqNAC73	C_Quinoa_Scaffold_2742	830479	831969
CqNAC74	C_Quinoa_Scaffold_2742	2425950	2427349
CqNAC75	C_Quinoa_Scaffold_2868	2813158	2817413
CqNAC76	C_Quinoa_Scaffold_1186	6601758	6605376
CqNAC77	C_Quinoa_Scaffold_2702	2127273	2129646
CqNAC78	C_Quinoa_Scaffold_3901	1027330	1037429

CqNAC79	C_Quinoa_Scaffold_1847	379048	380885
CqNAC80	C_Quinoa_Scaffold_4036	284851	288100
CqNAC81	C_Quinoa_Scaffold_3144	508634	514678
CqNAC82	C_Quinoa_Scaffold_1828	2728236	2729766
CqNAC83	C_Quinoa_Scaffold_3651	230705	236209
CqNAC84	C_Quinoa_Scaffold_2690	869497	871434
CqNAC85	C_Quinoa_Scaffold_2272	2995932	3001176
CqNAC86	C_Quinoa_Scaffold_1660	1227409	1230573
CqNAC87	C_Quinoa_Scaffold_3313	554469	558083
CqNAC88	C_Quinoa_Scaffold_2828	440003	448032
CqNAC89	C_Quinoa_Scaffold_2828	1153363	1157531
CqNAC90	C_Quinoa_Scaffold_2741	24164	26939

Table S5: PCR primers used for qRT-PCR in this study.

Gene name	Forward primer (5'-3')	Reverse primer (5'-3')
Actin	GGTATTGGAACGGTGCCAGT	GGACTCGTGGTGCATCTCAA
CqNAC16	AAGGAGAGTGGATCATGTAGCG	CCTTGTAGCCCTCTGGTTCA
CqNAC21	ACACGTGTCTAGGCTGCGGT	GAAGCACATTCCAAAAGCGG
CqNAC23	GGCAACTGATTACATCCCACAA	GCCAATTTGATTAAGATCATCAACAT
CqNAC32	TTACCCTCAGCTACCTCCCG	TCGACCTCAGCTATGATCGC
CqNAC36	TGACCGTTCATTCGCTTTGC	ATTCAGGTGCCGCGGTTAAC
CqNAC38	TTTCGCTTCCATCCGACTGA	CTCCAAATATCGCCTCGGCT
CqNAC42	ACTGATTACATCCCACAATG	TTAAGATCATGTTCAATCTCG
CqNAC45	ATTGAGCAGAGCAGAGGAGA	CGAGGTGAGAAGAAAAACCA
CqNAC62	CATGTGGAAGAATTCATCTG	AGCTAACGGGTAATTCTGAT
CqNAC66	GAGTGCGGCAAAGCCTACAA	ACCCGGTAAAGGCGAATTCA
CqNAC85	GGAGGTGTCATCGACGATCA	GGACGTCGCTTCTCAACCTT

Table S6: Orthology information of CqNACs and AtNACs from BLASTP.

CqNAC Name	AtNAC Name	Max Score	Total Score	Query Cover	E-Value	Per.ident
CqNAC36	RD26/AT4G27410	372	372	0.86	2.00E-130	75.20%
CqNAC66	RD26/AT4G27410	325	325	0.69	2.00E-111	65.20%
CqNAC36	ANAC019/AT1G52890	342	342	0.83	7.00E-118	67.20%
CqNAC66	ANAC019/AT1G52890	323	323	0.56	3.00E-110	85.71%
CqNAC32	ANAC2/AT3G15510	300	300	0.58	7.00E-100	74.26%
CqNAC38	ANAC2/AT3G15510	263	263	0.66	2.00E-85	61.80%
CqNAC23	NAC096/AT5G46590	283	283	0.79	1.00E-94	54.55%
CqNAC42	NAC096/AT5G46590	279	279	0.79	6.00E-93	54.55%
CqNAC21	ATAF1/AT1G01720	226	226	0.73	4.00E-73	52.21%
CqNAC16	NTL6/AT3G49530	247	283	0.52	3.00E-74	51.10%
CqNAC62	NTL6/AT3G49530	244	276	0.47	2.00E-73	57.51%
CqNAC45	NTL8/AT2G27300	224	224	0.7	2.00E-69	43.85%
CqNAC85	ANAC042/AT2G43000	223	223	0.74	5.00E-73	66.05%
